# Varicella-zoster virus reactivation from multiple ganglia: a case report

**DOI:** 10.4076/1752-1947-3-9134

**Published:** 2009-09-14

**Authors:** Mazyar Hashemilar, Kamyar Ghabili, Mohammadali Mohajel Shoja, Dariush Savadi-Oskouei, Hossein Keyvani

**Affiliations:** 1 Department of Neurology, Tabriz University (Medical Sciences), Tabriz, Iran; 2 Tuberculosis and Lung Diseases Research Center, Tabriz University (Medical Sciences), Tabriz, Iran; 3 Clarian Neuroscience Institute, Indianapolis Neurosurgical Group and Indiana University Department of Neurosurgery, Indianapolis, IN, USA; 4 Department of Virology, Iran University of Medical Sciences, Tehran, Iran

## Abstract

**Introduction:**

Simultaneous involvements of multiple cranial nerve ganglia (geniculate ganglion and peripheral ganglia of cranial nerves VIII, IX and X) by varicella-zoster virus and its subsequent activation may result in the characteristic eruptions of herpes zoster cephalicus. Coexistence of facial palsy and involvement of upper cervical dermatomes by varicella-zoster virus is quite rare.

**Case presentation:**

Here, we report a 71-year-old Iranian man with involvement of multiple sensory ganglia (geniculate ganglion and upper dorsal root ganglia) by varicella-zoster virus. He presented with right-sided facial weakness along with vesicular eruptions on the right side of his neck, and second and third cervical dermatomes.

**Conclusion:**

The present case is an example of herpes zoster cephalicus with cervical nerve involvement. Although resembling Ramsay Hunt syndrome with presence of facial nerve paralysis and accompanying vesicles, involvement of cervical dermatomes is not a feature of the classic Ramsay Hunt syndrome.

## Introduction

A wide spectrum of diseases including chickenpox and shingles can be induced by varicella-zoster virus (VZV) [1]. After the primary infection (chickenpox), the virus remains dormant in cranial nerves (e.g. geniculate ganglion of facial nerve) and dorsal root ganglia and then becomes reactivated decades later [1,2]. The reactivated VZV reaches the skin through axons usually causing pain and vesicular eruption restricted to a few dermatomes (herpes zoster or shingles) [2,3]. Subsequent to the involvement of sensory branches of facial nerve by VZV, the contiguous motor branches might become inflamed, resulting in facial palsy [4].

First noted by Ramsay Hunt in early nineteenths, simultaneous involvements of multiple cranial nerve ganglia (geniculate ganglion and peripheral ganglia of cranial nerves VIII, IX and X) by VZV and its subsequent activation may result in the characteristic eruptions of herpes zoster cephalicus [5,6]. Later in 1915, Sharpe classified herpes zoster cephalicus into five categories based on the inflammation of the geniculate, auditory, glossopharyngeal or vagal ganglia with or without the concomitant facial and acoustic symptoms [6]. Nonetheless, a few reports of the coexistence of facial palsy and involvement of upper cervical dermatomes by VZV can be read in the literature. Hereby, we report a patient with right-sided facial weakness along with vesicular eruptions on the right side of his neck and C2-C3 cervical dermatomes, indicating the involvement of multiple sensory ganglia (geniculate ganglion and upper dorsal root ganglia) by VZV.

## Case presentation

**Figure 1:**
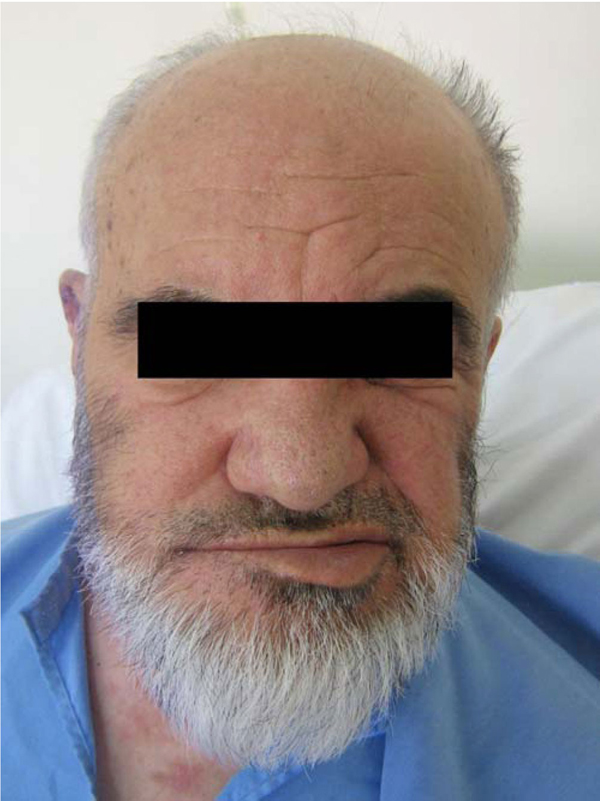
**Right facial nerve palsy**. Note the peripheral facial weakness.

**Figure 2:**
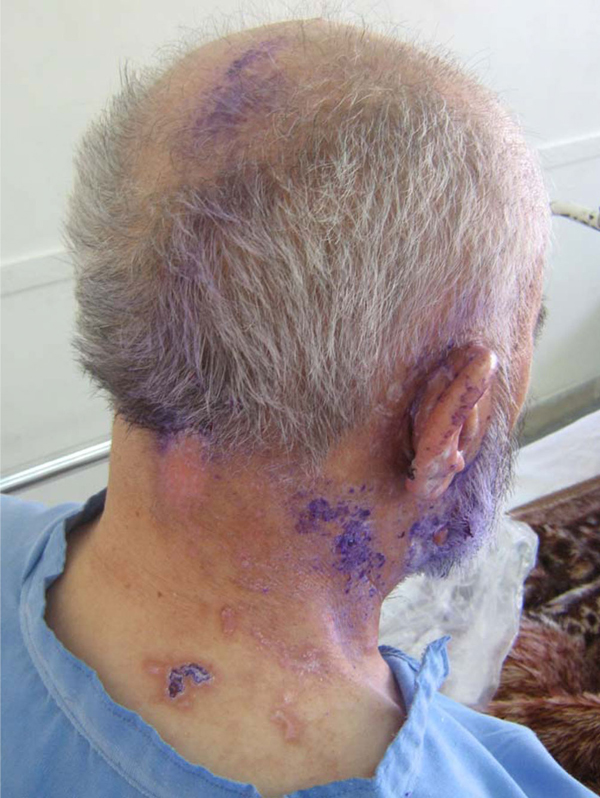
**Herpetic vesicles along the distribution of the right facial nerve and throughout the C2 and C3 dermatomes**.

A 71-year-old Iranian man developed severe right ear pain of three-week duration. He then developed a painful, vesicular eruption on the right side of his neck. With a presumptive diagnosis of herpes zoster reactivation, the patient was treated with oral acyclovir. However, he was re-admitted for an abrupt onset of facial weakness and mild vertigo. On examination, the patient had right-sided facial weakness (Figure 1). In addition, vesicular eruptions with adherent crusts and scabs (characteristic of VZV eruption) were noted within the right external auditory canal, over the mastoid, around the pinna, and C2-C3 cervical dermatomes (involvement of VII cranial nerve and C2-3 spinal nerves) (Figure 2). He had no associated immunocompromising condition including immunosuppressant drug use, leukemia, etc. A diagnosis of VZV reactivation from multiple ganglia was made based on the patient's characteristic presentation. The serum anti-VZV IgM antibody (ELISA) and VZV DNA (polymerase chain reaction) were negative. A computed tomography scan of the head was unremarkable. Further investigation revealed an increased white cell count (of 21600/μL) and a first hour erythrocyte sedimentation rate of 72 mm. The patient was placed on oral prednisone and oral acyclovir. A gradual improvement in facial weakness was noted. The herpetic vesicles on the head and neck were completely crusted. He was discharged with a favorable clinical condition.

## Discussion

VZV causes a wide range of disorders including chickenpox in childhood and shingles in elderly [1]. Once the chickenpox resolved, the virus settles down within the neurons of cranial nerves and dorsal root ganglia throughout the lifetime of the host [1,2]. A decline in host immunity, usually in elderly and immunocompromised individuals, results in reactivation of the virus from latency [3]. This is followed by the spread of reactivated virus to the skin through axons, causing a radicular pain and rash in the form of vesicles on an erythematous base with characteristic dermatomal distribution [1,2]. Since VZV is latent in numerous sensory ganglia, herpetic vesicles can occur anywhere on the body, commonly in thoracic, trigeminal and multiple dorsal root ganglia [2]. Exclusively, reactivation of VZV from the geniculate ganglion, nucleus of the sensory root of the facial nerve, can cause peripheral facial weakness as well as rash around the ear, known as Ramsay Hunt syndrome [4].

Concomitant involvement of multiple sensory ganglia by VZV was first noted by Hunt in 1910. He remarked the typical Ramsay Hunt syndrome along with the eighth nerve features including tinnitus, hearing loss, nausea and vertigo (as cited in reference [6]). Likewise, Sharpe [6] and Steffen and Selby [7] reported atypical cases of Ramsay Hunt syndrome in which upper cervical dermatomes and multiple cranial nerves were simultaneously involved. Nonetheless, the term "*Ramsay Hunt Syndrome*" is commonly believed to be used for those with involvement of the external auditory canal, but not for the cases with involvement of other cranial or cervical nerves or ganglia [8]. In this report, we described an elderly with right-sided facial weakness along with vesicular eruptions on the right side of his neck and C2-C3 cervical dermatomes, indicating involvement of multiple sensory ganglia (geniculate ganglion and upper dorsal root ganglia) by VZV.

The pathogenic mechanism of concurrent involvement of multiple sensory ganglia by VZV has been elucidated by several hypotheses [5]. Hunt first postulated an anatomic chain comprised by the geniculate, petrous, accessory, jugular, and C2-C3 dorsal root ganglia in which inflammation of a single ganglion could extend to proximate ganglia by contiguous anatomical contact [5,6]. Anatomically numerous interconnections have been denoted between the facial nerve and VIII, IX, X, XI, XII and upper cervical nerves. The anastomoses between the lower cranial and upper cervical nerves are referred to as spinal accessory nerve plexus; this plexus shows high individual variations [9]. Hence, the communications between the facial nerve and other nerves of the head and neck region may explain the simultaneous involvement of cranial and cervical nerves by reactivated, spreading VZV [10]. Another dissemination route of VZV is surmised to be the simultaneous reactivation of VZV in multiple ganglia and inter-connecting nerves [11]. This theory is supported by the report of Gilden and colleagues who found VZV DNA in multiple cranial nerves, dorsal root ganglia and celiac ganglia [12]. Some also believe that VZV-induced cranial polyneuropathies occur by the spread of a virus through a common blood supply [1]. In the present case, we surmise that spreading of reactivated VZV via the anatomic continuity caused multiple sensory ganglia involvement including geniculate ganglion and C2-C3 dorsal root ganglia.

Shingles is usually diagnosed by inspection of an asymmetrical dermatomal rash and synchronous occurrence of skin lesions (erythema, vesicular, pustular and finally crustous lesions) [13]. In addition, polymerase chain reaction (PCR) and the appropriate serologic assays (VZV IgM and IgA antibodies) on CSF, serum and vesicular fluid may also detect VZV infection [13,14]. However, antibody assays may have little diagnostic yield because of persistence of anti-VZV antibodies in the serum of nearly all adults [15]. In the present patient, the serum IgM antibody titer to VZV on ELISA was negative, which might denote the quite long interval from the onset of the herpetic vesicles to the beginning of the facial palsy.

## Conclusion

In summary, the present case is an example of herpes zoster cephalicus with cervical nerve involvement. Although resembling Ramsay Hunt syndrome with presence of facial nerve paralysis and accompanying vesicles, involvement of cervical dermatomes is quite rare and is not a feature of the classic Ramsay Hunt syndrome.

## Abbreviations

CSF: cerebrospinal fluid; DNA: deoxyribonucleic acid; ELISA: enzyme-linked immunosorbent assay; IgA: immunoglobulin A; IgM: immunoglobulin M; PCR: polymerase chain reaction; VZV: varicella-zoster virus.

## Consent

Written informed consent was obtained from the patient for publication of this case report and accompanying images. A copy of the written consent is available for review by the Editor-in-Chief of this journal.

## Competing interests

The authors declare that they have no any competing interests.

## Authors' contributions

MH and DSO contributed to acquisition of data and interpreted experiments. KG and MMS interpreted experiments and revised the manuscript. HK performed ELISA and PCR tests and helped to draft the manuscript. All authors read and approved the final manuscript.
